# Data anomalies and apparent reporting errors in ‘Randomized controlled trial testing weight loss and abdominal obesity outcomes of moxibustion’

**DOI:** 10.1186/s12938-020-0753-z

**Published:** 2020-02-18

**Authors:** Luis M. Mestre, Stephanie L. Dickinson, Lilian Golzarri-Arroyo, Andrew W. Brown, David B. Allison

**Affiliations:** 1 Department of Epidemiology and Biostatistics, Indiana University School of Public Health-Bloomington, 1025 E 7th St, Bloomington, IN 47405 USA; 2 Department of Applied Health Science, Indiana University School of Public Health-Bloomington, 1025 E 7th St, Bloomington, IN 47405 USA

**Keywords:** Randomization, Clinical trials, Moxibustion therapy, Weight reduction, Waist circumference, Waist-to-hip ratio

## Abstract

Randomized Controlled Trials (RCTs) are the best method to determine causal effects for treatments if they are well done and well reported. Good evidence about proposed treatments for obesity is needed, and Hsieh et al. (Biomed Eng Online 17:149, 2018) are to be commended for putting moxibustion to the test. However, careful evaluation of the paper reveals inconsistencies and apparent reporting errors, which raise doubts about conclusions from the study.

Randomized controlled trials (RCTs) are the best method to determine causal effects for treatments if they are well done and well reported. Good evidence about proposed treatments for obesity is needed, and Hsieh et al. [1] are to be commended for putting moxibustion to the test. However, careful evaluation of the paper, similar to a prior review of another paper on moxibustion [2], reveals inconsistencies and apparent reporting errors, which raise doubts about conclusions from the study.

The primary concern relates to the randomization process. Table 1 in Hsieh et al., which describes baseline anthropometrics of both groups in their study, showed that baseline body weight (BW) differed with a high degree of statistical significance (*p* < 0.001) based on the comparison of groups using a *t* test [1], which, by definition, is very unlikely to occur under randomization [3–6]. We also note that the baseline variances for BW were significantly heterogeneous (Bartlett’s test; $\chi^{2} = 6.86, \;df = 1, \;p = 0.0088$), which is again, by definition, highly unlikely under randomization. These baseline distribution differences by group raise questions as to whether the randomization was properly performed. Results might be influenced by any problem in the randomization process, which could bias the estimated treatment effects [6]. The details of the randomization process were not clear, and we found no clinical trial registration for the study; randomization details should be reported as per the CONSORT statement [7].

A collection of secondary concerns, unrelated to our core concern of randomization, comprises some inconsistencies and errors found in the paper that affect the understanding of the study design and confidence in the results. First, Figure 1 of Hsieh et al. indicates “Discontinued Intervention = 30” of the control group for follow-up, which should presumably be zero because other details in the paper indicate the control group sample size is *n* = 30 enrolled and *n* = 26 analyzed. Second, the third paragraph of the Results section indicates a *p*-value of 0.002 for BW and 0.003 for WC, whereas Table 1 reports the *p*-values as 0.0002 and 0.0003, respectively. Third, Tables 3 and 4 show the results of the WC and WHR in kg units instead of cm for Table 3 and unitless for the ratio in Table 4. Finally, we note that error terms are missing for some slopes but not others in Tables 2–4.

Although the lack of an attention-placebo control group matched for amount and type of study staff contact limits the ability to determine whether it is the treatment per se that is responsible for any effects of treatment assignment (if there are any such effects), we have chosen to focus here on the issue of proper randomization, without which there cannot be any determination of effects at all, let alone their mechanism.

We believe these anomalies should be corrected or explained, particularly with respect to the unlikely baseline imbalances, which raise concerns about the randomization process.

## Data Availability

Not applicable.

## Abbreviations

BW: Body weight
CONSORT: Consolidated standards of reporting trials
RCTs: Randomized clinical trials
WHR: Waist-to-hip ratio
WC: Waist circumference
